# MicroRNAs Based Therapy of Hypertrophic Cardiomyopathy: The Road Traveled So Far

**DOI:** 10.1155/2015/983290

**Published:** 2015-10-04

**Authors:** Catarina Roma-Rodrigues, Luís R. Raposo, Alexandra R. Fernandes

**Affiliations:** ^1^UCIBIO, Departamento de Ciências da Vida, Faculdade de Ciências e Tecnologia da Universidade Nova de Lisboa, Campus de Caparica, 2829-516 Caparica, Portugal; ^2^Centro de Química Estrutural, Complexo 1, Instituto Superior Técnico, Universidade de Lisboa, Avenida Rovisco Pais, 1049-001 Lisboa, Portugal

## Abstract

Hypertrophic cardiomyopathy (HCM) is an autosomal dominant disease characterized by variable expressivity, age penetrance, and a high heterogeneity. The transcriptional profile (miRNAs, mRNAs), epigenetic modifications, and posttranslational modifications seem to be highly relevant for the onset of the disease. miRNAs, small noncoding RNAs with 22 nucleotides, have been implicated in the regulation of cardiomyocyte function, being differentially expressed in several heart diseases, including HCM. Moreover, a different miRNA expression profile in the various stages of HCM development is also observed. This review summarizes the current knowledge of the profile of miRNAs characteristic of asymptomatic to overt HCM patients, discussing alongside their potential use for diagnosis and therapy. Indeed, the stability and specificity of miRNAs make them suitable targets for use as biomarkers for diagnosis and prognosis and as therapeutical targets.

## 1. Introduction

Hypertrophic cardiomyopathy (HCM) is the most common familial form of cardiomyopathies, occurring mainly due to mutations in genes encoding for the cardiac contractile apparatus [1, 2]. Among the 1,400 mutations identified has responsible for HCM, 70% of them are in sarcomere genes cardiac *β*-myosin heavy chain (*MYH7*) and cardiac myosin binding protein C (*MYBPC3*) [3]. Mutations are inherited in an autosomal dominant pattern affecting 1 : 500 individuals in the general population, with 60% of the sarcomere gene mutations being described as familial HCM [1, 4]. The progression of symptoms in HCM is not straightforward, due to morphological and pathological heterogeneity, age dependency and incomplete penetrance, resulting in prognosis uncertainty. Accordingly, the clinical outcome of HCM is diverse, ranging from asymptomatic patients to cardiac arrhythmias, congestive heart failure, and sudden cardiac death [5]. A high percentage of patients are asymptomatic or mildly symptomatic and diagnosis is made during family screening or incidentally by the observation* via* conventional echocardiography of an unexplained left ventricular wall thickening [6]. Due to the heterogeneity of the pathology, the diagnosis is made in the middle or late adulthood when the morphology and functional debility of the heart have already progressed [6]. This is particularly devastating for asymptomatic or mildly symptomatic young patients that may experience sudden cardiac death [7].

One of the most striking features of HCM is the inexistence of a correlation between genotype and phenotype, has family members carrying the same mutation developed distinct symptoms [4, 8, 9]. The clinical outcome of HCM is likely to be the sum of genetic mutations with age-related decline in protein-protective mechanisms and environmental factors such as lifestyle, degree of physical exercise, and blood pressure [10, 11]. In this regard, the transcriptional profile, epigenetic modifications, and protein posttranslational modifications seem to be crucial to the events in the cardiomyocyte that will trigger the onset of HCM [11, 12]. In the last decade microRNAs (miRNAs), small noncoding endogenous RNAs that regulate gene expression by directing their target mRNAs for degradation or translational repression, were revealed as important regulators of the heart physiology [12–15], with a characteristic expression profile in different cardiovascular diseases [16–18]. The small size (22 nucleotides in length) and stability make miRNAs suitable targets for silencing by antisense oligonucleotides or by restoring their function by using synthetic double stranded miRNAs or viral vector based overexpression [16, 19]. Moreover, the discovery of the presence of miRNAs in the bloodstream [20] highlighted the possibility of their use as circulating biomarkers for HCM. However, the role of miRNAs in the progress of HCM is still not completely understood. In this review the possibility of the use of miRNAs based therapy throughout the course of events occurring in the path from asymptomatic to overt HCM will be discussed.

## 2. MicroRNAs

Since their discovery in 1993, miRNAs have been increasingly recognized as an important class of regulatory small noncoding RNAs that function as negative regulators of gene expression [17, 21]. Approximately 60% of protein coding genes are regulated by miRNAs [22]. Concerning the function of miRNAs, some act as key regulators of a particular cellular process, affecting the expression of hundreds of genes simultaneously, while others may regulate specific individual mRNA targets or regulate target mRNAs cooperatively [22]. These miRNA regulatory networks are important in the “fine tuning” of the overall protein expression in cells and are important in the cellular responses to stress [23].

**Figure 1:**
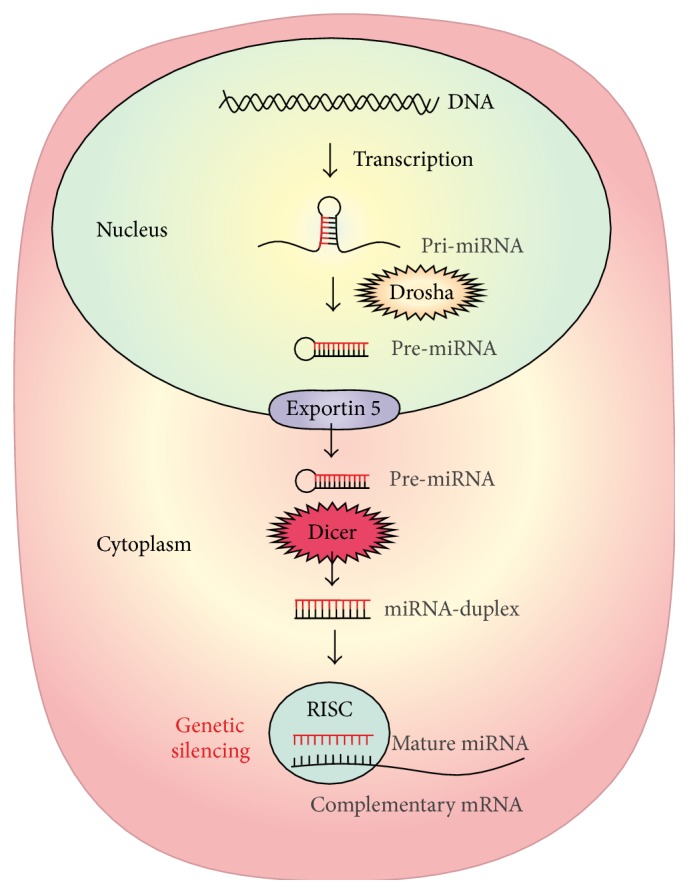
Schematic representation of miRNA biogenesis and function. The biogenesis of miRNAs is initiated in the nucleus by the transcription of pri-miRNAs that, after hydrolyzation mediated by the RNAse Drosha, form pre-miRNA. After transport to the cytoplasm mediated by exportin 5, the loop of pre-miRNAs is cleaved by Dicer RNAse, forming the miRNA-duplex, which is incorporated in the RISC complex. The miRNA-duplex is then separated forming the mature miRNA that will inhibit translation by base pairing with the 3′UTR of the target mRNA.

miRNAs are transcribed by RNA polymerases II and III from different genomic locations [24–27] and can be located in introns of protein coding genes, such as the miR-25-miR-93-miR-106b cluster in an intron of the* MCM7* gene, or in exons of protein coding genes, as miR-985 located in the last exon of* CACNG8* gene [27]. miRNAs are also transcribed from introns of protein noncoding genes such as the miR-15a-miR-16-1 cluster in an intron of the* DLEU2* gene and from exons of noncoding genes such as miR-155 coded by an exon of* BIC* gene [27]. Pri-miRNA are long molecules (more than 1 Kbp) that fold into a characteristic secondary structure, comprising a terminal loop, a stem of approximately 33 base pairs (bp), and flanking segments of single stranded RNA [27]. In the nucleus, the microprocessor complex which contains Drosha RNase cleaves the pri-miRNAs 11 bp away from the junction of single stranded RNA with double stranded RNA [28, 29] (Figure 1). This cleavage generates precursor miRNAs (pre-miRNAs) that maintain the stem-loop conformation [30]. Pre-miRNAs are then exported from the nucleus to the cytoplasm by the RanGTP-binding export receptor, exportin 5 [31] (Figure 1). In the cytoplasm, the loops from pre-miRNAs are then cleaved by Dicer RNase, thus forming the mature double stranded miRNA with 22 nt [16, 32, 33] (Figure 1). These miRNAs are incorporated as single stranded RNAs into the RNA-induced silencing complex, RISC [30] (Figure 1). Genetic silencing is accomplished by base pairing between the seed region (nucleotides 2–8 in the 5′ region) of the miRNA in the RISC complex and the complementary region in the 3′-untranslated region of the target mRNAs [34].

## 3. miRNAs in Hypertrophic Cardiomyopathy

Perturbations of the sarcomere function due to HCM related mutations result in an energy deficiency that ultimately compromise the relaxation capacity of the cardiomyocyte and consequently the force of contractibility [35, 36]. These events will develop distinct cardiac histopathological features, such as cardiomyocytes hypertrophy, which are indicators of an early stage of HCM pathology [7, 37]. Further events that precede cardiomyocyte hypertrophy include myocyte disarray, consisting in an asymmetric distribution of the hypertrophic cardiomyocytes within the ventricle, and interstitial fibrosis, mainly due to an increased synthesis of collagen as a consequence of sarcomere mutations and accumulation of fibroblasts after cardiomyocyte death [3, 37, 38]. This maladaptive cardiac remodeling contributes to the development of HCM pathology, such as left ventricular (LV) hypertrophy and arrhythmias, and subsequent appearance of several clinical manifestations, such as obstruction of the outflow tract in the LV or LV systolic dysfunction, which ultimately result in poorer prognosis due to an increased risk of heart failure or sudden death [3, 7, 37, 38]. The morphological heterogeneity and incomplete penetrance of HCM render the study of the pathology progression and the establishment of an analogy between mutation and miRNA profile difficult. Nevertheless, several miRNAs were described as being important regulators of the cardiomyocytes hypertrophy and fibrosis [39], and a different miRNA expression profile in ventricle cardiomyocytes between the early and end stages of HCM pathology was described [40]. Hence, three major phases can be considered in HCM progression as follows: (i) Asymptomatic phase, considered as the early stage of the pathology where no major pathophysiological changes have been developed; (ii) mildly asymptomatic phase, considered as the onset of the disease where the cardiomyocyte relaxation ability is compromised resulting in an expansion of cardiomyocyte hypertrophy and myocardium fibrosis; and (iii) overt HCM phase, which is the symptomatic stage of HCM, characterized by overt LV hypertrophy and arrhythmias.  Table 1 resumes the differential expression profile of miRNAs throughout the course of HCM pathology and the following chapters will discuss their possible use in the diagnostics and prognostics of HCM phenotype.

### 3.1. Asymptomatic HCM

Due to obvious limitations in the study of asymptomatic patients, no studies have been published regarding the miRNA profile during the asymptomatic stage of HCM related mutations carriers. However, a recent study of Bagnall and coworkers [40] revealed a differential miRNA expression profile since the predisease state of the animals (after 5 days). In this study, the miRNA profile of the ventricles of a transgenic HCM double mutant mouse model suggested downregulation of miR-1 and miR-133 in a primary stage of the disease prior to a pathophysiological change [40]. Interestingly, the expression profile of both miRNAs seems to be maintained throughout the course of HCM progression (Table 1) [40, 42, 46]. Downregulation of miR-1 is a well-described event that occurs during cardiomyocyte hypertrophy [46–50], which is a response of the terminally differentiated cardiomyocytes to mechanical or pathological stress, consisting in an increase in cell size and concomitant reinduction of the fetal gene program [51]. While miR-1 is highly abundant in terminally differentiated cardiomyocytes, its level is lower in the developing embryonic hearts of mice and hypertrophic cardiomyocytes [46, 49].

Despite the tempting suggestion for the use of miR-1 and miR-133 as biomarkers to identify subjects who are at risk of the development of HCM symptoms and/or as therapeutic targets, this will only be conceivable if the alteration of abundance of these miRNAs in asymptomatic patients can be identified through minimal-invasive procedures. Interestingly, an increased circulating concentration of miR-1 and miR-133 in patients with overt HCM was registered [15, 52]. The contradictory higher levels of circulating miR-1 and miR-133 in HCM patients and lower concentration in hypertrophic cardiomyocytes reflect ischemic episodes and consequent cardiomyocyte death [42]. Because of the irreversible mutation-induced perturbations of the cardiomyocyte structure and function established in the postnatal period [53] it is plausible that ischemic death might occur in a higher frequency throughout life of an HCM patient. Hence, an increased concentration of circulating miR-1 and miR-133 even in asymptomatic patients can be hypothesized.

### 3.2. Mildly Asymptomatic HCM

The knowledge of regulation of cardiac hypertrophy and fibrosis mediated by miRNAs, particularly in HCM related pathologies, represents novel signatures of disease that can be targeted for restraining clinical phenotypes [15, 39, 54]. Gain-of-function/loss-of-function approaches* in vitro* using cardiomyocytes cultures and* in vivo* studies using mutant mouse heart as a model allowed taking insights into the role of miRNAs in the mechanisms involved in HCM development and revealing miRNAs that can be used as biomarkers of different traits of the pathology. miRNAs involved in cardiomyocyte hypertrophy can be classified as being prohypertrophic and antihypertrophic, if the mechanisms they regulate increase or decrease the degree of hypertrophy [55]. Examples of prohypertrophic miRNAs are miR-23a [56] and miR-499 [18], while miR-22 [57], miR-26b [58], miR-451 [43], and miR-98/let-7 [59] are antihypertrophic miRNAs.

### 3.3. Overt HCM

Efforts have been made in order to find the relation between HCM related mutations and cardiomyocyte miRNA profile. However, they are limited by the use of animal models or by studies performed only in an advanced state of HCM, which is mainly due to the invasive procedures for sampling.

Nevertheless, a differential miRNA expression profile induced by HCM pathologies was observed by Kuster and coworkers [44] that analyzed the miRNA expression profile in affected heart tissues of patients carrying mutations in* MYBPC3* and by Palacín and coworkers [45], which, inclusively, were able to find a different miRNA profile between the hearts of HCM patients with distinct mutations (Table 1). Furthermore, Vignier and coworkers [60] suggested a distinct circulating miRNA profile between cardiovascular ischemic pathologies and cMyBP-C related cardiomyopathy.

There are some limitations imposed by the current murine models used for HCM studies. Although animal models can provide information about the release kinetics of miRNAs, these findings cannot be directly transferred to humans, because of the different physiological parameters and species specific differences in miRNA expression. However, despite the species specific differences, several miRNAs were found to be equally regulated in murine and humans with overt HCM (Table 1), with upregulation of miR-21, miR-132, and miR-222 and downregulation of miR-1, miR-30b, miR-133b and miR-150.

### 3.4. Circulating miRNAs in HCM

One of the most exciting possibilities of the characterization of circulating miRNA expression profile is its use as biomarkers for diagnosis and prognosis. Indeed, circulating miRNAs fulfill several criteria that make them suitable for use as clinical biomarkers, such as accessibility through minimal-invasive procedures, a long half-life within the sample, possibility of a rapid and accurate detection, high degree of specificity and sensitivity, and ability to differentiate pathologies [61]. Interestingly, studies of the miRNA profile of the plasma of HCM patients performed by Roncarati and coworkers [15] suggested a disease specific profile that distinguished between HCM and aortic sclerosis hypertrophies. These authors found that 3 miRNAs (miR-199a-5p, miR-199a-27a, and miR-199a-29a) correlated with hypertrophy but only miR-29a is significantly associated with both hypertrophy and fibrosis, identifying it as a potential biomarker for myocardial remodeling assessment in HCM [15]. However, further studies are needed that may reveal an HCM specific circulating miRNA profile that could distinguish primary HCM even before development of symptoms. The quality and integrity of RNA extracted from hearts or biological fluids are fundamental for miRNA profiling based on microarrays and real-time PCR. This is a destructive testing that limits the number of samples available for the HCM studies. Differences in the sample quality and integrity may justify some discrepancies between studies.

Recently, gold nanobeacons have been proven useful tools for diagnostics [62]. Due to the design of an antimiR oligonucleotide bound to a gold nanoparticle it was possible to detect the hybridization of the miR-21 to its respective antimiR due to an increase of the gold nanobeacons' fluorescence [63]. This methodology allows simultaneously the detection of a specific miRNA directly on a blood sample without the need of disrupting cells for RNA extraction and at the same time permits a specific silencing [63]. These gold nanobeacons have also the potential to be further functionalized with specific targeting moieties (e.g., antibodies) in order to achieve specific targeting [64]. The application of these nanoformulations for simultaneous detection and silencing of miRNAs directly from blood samples can overcome the bias introduced by sample quality and integrity that may justify some discrepancies between studies, opening a new avenue for future early diagnosis, prognosis, and therapy in hypertrophic cardiomyopathy.

## 4. Therapies Based on miRNAs

The ability of miRNAs to be naturally transported in biological fluids and delivered to cells makes them good targets for therapy in the context of cardiomyopathy [23, 65].

Therapies based on miRNAs involve the restoration of miRNA function that has been done through the use of synthesized miRNA-duplexes (miRNA-mimics), which are double stranded oligonucleotides including the mature miRNA sequence and the complementary passenger strand [19, 65]. The passenger strand can be chemically modified, with cholesterol, for instance, to improve cellular uptake [19]. Targeting of these miRNA-mimics can be augmented through the use of lenti-, adeno-, or adeno-associated viruses (AAV) [19, 20, 65]. On the other side, miRNAs silencing has been accomplished by the use of synthetic oligonucleotides with 8–25 nt of length, complementary to the seed sequence of the miRNA of interest, called antimiRs [19, 65]. Chemically modified antimiRs have been successfully used in order to increase their binding affinity to the target miRNA, biological stability, and pharmacokinetic properties [19]. The antagomiRs are a class of antimiRs in which the oligonucleotides are conjugated with cholesterol [65]. Other frequent chemical modifications of the antimiRs include 2-O-methyl-modified oligonucleotides and locked nucleic acid- (LNA-) modified oligonucleotides [19, 65].

HCM therapeutics based on miRNAs involves the modulation of expression of these noncoding RNAs in cell, by inactivating the function of prohypertrophic miRNAs using synthetic miRNAs or by using miRNAs mimics oligonucleotides of antihypertrophic miRNAs [19].

The possibility of the use of miRNAs based therapy in treatment of HCM is highlighted by several* in vivo* studies [46, 66, 67] that were able to inhibit cardiomyocyte hypertrophy and fibrosis (Table 2). With the purpose to study the effect of miR-133 in the cardiomyocyte hypertrophy, Carè and coworkers [46] successfully used a miR-133 RNA-mimic combined with an adenoviral vector and an antagomiR in the hearts of a mouse model of AKT induced heart hypertrophy and in a normal C57BL/6 mouse line, respectively. Similarly, Thum and coworkers [66] and Ucar and coworkers [67] were able to inhibit cardiomyocyte hypertrophy using an antagomiR for miR-21 and for miR-132, respectively, in mice models with heart failure induced by pressure overload of the left ventricle. An antimiR for miR-208a was successfully used to reduce miR-208a expression in a rat model of diastolic heart failure [68]. However, possibly due to the limited knowledge on HCM progression, miRNAs based therapies are yet to be made.

Despite the promising use of therapy based miRNAs, modulation of the function of miRNAs raises several concerns. The uptake of miRNA-mimics can result not only in the restoration of miRNA function in affected cells but also in the overexpression of the miRNA in other cells. In the same way, the inhibition of miRNA activity by antimiRs can be done on off-target locations. Therefore, targeting would be important in order to meliorate miRNA therapy, which can be accomplished with nanotherapies [62]. Furthermore, the alteration of systemic levels of miRNAs can perturb the homeostasis of circulating miRNA and disturb normal functions in cells and tissues and thus create unwanted side effects.

## 5. Conclusions

The study of miRNAs expression profiles that will allow its use for diagnosis, prognosis, and therapeutics of HCM is still in its infancy. The high heterogeneity of HCM pathology renders the detection of a disease specific miRNA profile difficult. Furthermore, due to the invasive procedures required for human cardiomyocytes analysis, the analysis of the cardiac miRNA profile is only possible when the disease reached a terminal state. The use of murine models has proven to be fruitful to study the several stages of the disease. Nanobiotechnology allows the simultaneous detection and silencing of miRNAs directly from blood samples will speed up early diagnosis, prognosis, and therapy in hypertrophic cardiomyopathy.

## Figures and Tables

**Table 1:** miRNA expression profile in the development of hypertrophic cardiomyopathy (HCM). The development of the pathology is associated with the stress imposed to cardiomyocytes due to mutations in genes of the cardiac contractile apparatus. The passage from asymptomatic to mildly asymptomatic stages is related to the heart morphology (schematically represented). In mildly asymptomatic patients, a cardiac remodeling, consisting in cardiomyocyte (red cells) hypertrophy, fibrosis mediated by an increased synthesis of interstitial collagen, cardiomyocytes spatial misalignment, and substitution of dead cardiomyocytes by fibroblasts (blue cells), is performed [3, 41]. Overt HCM is characterized by a cardiac left ventricular hypertrophy higher than 15 mm. miRNAs whose expression is consistently altered in tissues and in circulation are in bold.

	Murine heart	Human heart		Circulating miRNAs
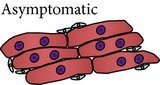	*Downregulated *			
	**miR-1** [40] miR-133 [40]			
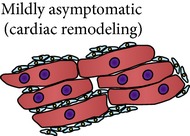	Upregulated			*Upregulated* *Fibrosis* miR-29a [15] *Cardiomyocyte* *hypertrophy* miR-27a [15] miR-29a [15] miR-199a-5p [15] *Other functions* **miR-21** [15] miR-26a [15] miR-30a [15] miR-126-3p [15] miR-133a [15] miR-143 [15] miR-145 [15] miR-155 [15] miR-199a-3p [15] miR-483-5p [42]
	**miR-21** [40] miR-132 [40] miR-214 [40] miR-331 [40]			
	*Downregulated *			
	**miR-1** [40]			
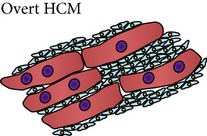	*Upregulated *	*Upregulated *		
	**miR-21** [40] miR-31 [40] miR-34b-3p [40] miR-132 [40] miR-142 [40] miR-214 [40] miR-222 [40]	**miR-21** [43] miR-34b^*^ [44] miR-92a [45] miR-96 [44] miR-130b [43] miR-132 [44] miR-181a-2^*^ [44] miR-184 [44]	miR-204 [44] miR-222^*^ [44] miR-383 [44] miR-371-3p [44] miR-497 [44] miR-590-5p [45] miR-708 [44]	
	*Downregulated *	*Downregulated *		
	**miR-1** [40] miR-30b-5p [40] miR-30c [40] miR-30e [40] miR-133a [40] miR-133b [40] miR-150 [40] miR-486-5p [40]	**miR-1** [45] miR-10b [44] miR-10a [44] miR-10b^*^ [44] miR-30b [45] miR-133b [45] miR-139-5p [43] miR-139-3p [43] miR-144^*^ [43] miR-150 [43] miR-191 [45]	miR-208b [45] miR-218 [45] miR-363 [43] miR-374 [45] miR-451 [43] miR-454 [45] miR-486-3p [43] miR-495 [45] miR-1246 [43] miR-3141 [43]	

miR-X^*^: antisense miRNA star.

**Table 2:** Resume of miRNAs based therapies targeting cardiomyocytes hypertrophy and fibrosis. The oligonucleotide modification, miRNA, the model used in the study, and the obtained result are depicted. LNA: locked nucleic acid.

	Oligonucleotide modification	miRNA	Model	Result	Ref.
AntimiR	AntagomiR	miR-133	C57BL/6 mice	Repression resulted in cardiac hypertrophic phenotype	[46]
	AntagomiR	miR-21	Heart failure induced by pressure overload mice	Repression reduces cardiac ERK-MAP kinase activity, inhibits interstitial fibrosis, and attenuates cardiac dysfunction	[66]
	AntagomiR	miR-132	Heart failure induced by pressure overload mice	Repression rescues heart hypertrophic phenotype	[67]
	LNA-modified oligonucleotide	miR-208a	Diastolic heart failure rats	Repression resulted in reduction of cardiac remodeling	[68]

RNA-mimic	Adenoviral vector containing miR-133a-2 precursor sequence	miR-133	AKT induced heart hypertrophy mice	Overexpression resulted in attenuation of cardiac hypertrophic phenotype	[46]
